# Water, Sanitation, and Hygiene Practices in Urban Slums of Eastern India

**DOI:** 10.1093/infdis/jiab354

**Published:** 2021-11-23

**Authors:** Suman Kanungo, Pranab Chatterjee, Jayanta Saha, Tania Pan, Nandini Datta Chakrabarty, Shanta Dutta

**Affiliations:** 1 Division of Epidemiology, National Institute of Cholera and Enteric Diseases, Kolkata, India; 2 Department of International Health, Johns Hopkins University Bloomberg School of Public Health, Baltimore, Maryland, USA

**Keywords:** sanitation, drinking water, hygiene, enteric fever, typhoid

## Abstract

**Background:**

The Sustainable Development Goals identified universal access to water and sanitation facilities as key components for improving health. We assessed water, sanitation, and hygiene (WASH) practices and associated determinants among residents of urban slums in Kolkata, India.

**Methods:**

Information on WASH practices was collected in 2 surveys (2018 and 2019) from participants of a prospective enteric fever surveillance conducted in 2 municipal wards of Kolkata. A composite WASH practice score was computed and a hierarchical stepwise multiple linear regression model constructed to identify key determinants of improved WASH score.

**Results:**

Over 90% of households had access to piped water; 6% reported access to continuous supply. Adult women (61% in 2018; 45% in 2019) spent 20 minutes daily to fetch water. Access to improved latrines was almost universal, although 80% used shared facilities. Unhealthy disposal of children’s stools was reported in both rounds. Food hygiene practices were high, with most (>90%) washing uncooked items before eating; frequent consumption of street food items was reported.

**Conclusions:**

The study area reported high WASH coverage. Unhygienic behavioral patterns predisposing to food- or water-borne diseases were also noted. Awareness building and sustainable community mobilization for food hygiene needs to be emphasized to ensure community well-being.

Water, sanitation, and hygiene (WASH) remain critically important to public health. Lack of access to safe water, proper sanitation, and inadequate hygiene practices have been linked with the increased risk of transmitting infectious diseases like cholera, typhoid, hepatitis A, and many other water-related diseases [1, 2]. Updated analysis of World Health Organization (WHO) data indicate that globally an estimated 8 29 000 deaths and 49.8 million disability adjusted life years, with a mortality rate of 11.7 deaths per 100 000 population in 2016, could be attributed to unsafe WASH practices [3–6]. The highest mortality rates associated with inadequate WASH are seen in low- and middle-income countries of South-East Asia and Africa, with 15.4 and 45.8 deaths per 100 000, respectively [7].

India has an enormous burden of enteric infections such as diarrhea and enteric fever, as a result of unabated population growth, high levels of poverty and illiteracy, and inequity in access to safe drinking water and sanitation services [8]. However, the provision of safe water and adequate sanitation does not always ensure hygienic use or adoption of other sanitary practices, as it has been demonstrated that point-of-use contamination of drinking water, obtained from safe, municipal sources, is a major determinant of diarrheal disease occurrence in children in urban slums of India [9]. The Swachh Bharat Abhiyan (Clean India Mission) has resulted in major improvements in access to safe potable water, sanitation, and hygiene facilities, especially in urban India. According to the statistics released by the Joint Monitoring Program for Water Supply, Sanitation, and Hygiene in December 2019, between 2000 and 2017, access to basic safe water has gone up from 94.7% to 96.3%, access to basic sanitation facilities has gone up from 49.3% to 72%, and around 80% of the urban population have been covered by basic hygiene services since 2012, when these estimates were first reported [10]. However, despite incremental access to improved WASH amenities, thanks to the Swachh Bharat Abhiyan, pockets of inequity still remain [11]. These inequities are especially pronounced in urban slums, where population densities are high, access to services are low, and as a consequence of poverty, adverse social determinants, and biological factors playing synergistic negative roles, the levels of morbidity and mortality are unacceptably high.

Enteric fever is a condition that imposes a significant public health burden and is a cause for concern in India, with varied geographic heterogeneity across the country [12]. Typhoid causes an estimated 235 cases per 100 000 persons per year in Kolkata, as reported from cohort studies in urban slum dwellers [13]. To generate evidence and current estimates of enteric fever burden in India, fever surveillance was conducted in Kolkata as a part of the National Surveillance System for Enteric Fever in India (NSSEFI) [14]. Individual household WASH services and practices were assessed at baseline and at 1 year of follow-up. This study reports on the gaps in WASH conditions in urban slums of Kolkata to inform and augment appropriate future interventions in the region.

## METHODS

### Study Area and Population

Kolkata is one of the most populous cities of India, with 19 million people living in its central and suburban areas, which consists of 144 administrative wards. The study area consisted of 2 such wards, numbers 58 and 59, in the eastern part of the city. A large proportion of the residential areas in these localities are densely populated urban slum settlements. These are officially recognized and registered slums, with narrow streets and lanes characterized by overcrowding, shared intermittent water supply, shared community latrines, and inadequate sewage disposal through open drains.

### Study Process

A community-based prospective enteric fever surveillance was initiated in November 2017 and it continued up to December 2019, recruiting children aged 6 months to younger than 15 years residing in the area. Five field health outposts, staffed by physicians and health workers, were set up in the 2 wards for enteric fever surveillance. Eligible children were enrolled prospectively following appropriate consent/assent processes. All enrolled children underwent active weekly inspection either through personal home visits or through telephone interviews for 24 months or until they attained 15 years of age, whichever was earlier. Any episode of fever (≥38°C/100.4°F) among the children included in the cohort was reported to the study team by the caregiver, and details recorded in the fever case report form. A blood sample was collected for culture from every subject with a history of fever for 3 consecutive days. Detailed methodology of the study has been reported previously [14].

A pretested questionnaire was administered to collect information on demography, assets, socioeconomic status, cooking fuel, access to safe water, sanitation, and hygiene practices from the households at the baseline (March to September 2018) and again 1 year later (March to September 2019). We followed the Joint Monitoring Program 2017 definitions for improved sources of drinking water and sanitation [15].

### Data Analysis

A custom-designed data collection application, developed by the central data management team, was used for coded digital data collection in the field using an android-based handheld tablet computer. Rigorous checking and verification of the collected data, including quality control of the data, were carried out on a regular basis through the central data management team. All the recorded information was compiled and managed in a database locally at the Indian Council of Medical Research (ICMR)-National Institute of Cholera and Enteric Diseases (NICED). All data analysis was done using Stata version 16.1 (Stata Corp). The characteristics of the households being surveyed and reported WASH practices were described by the 2 rounds of the survey. The WASH information was used to develop a composite WASH score to summarize the WASH risks of the households and the difference in the score between the 2 rounds was also computed. Univariable linear regression analyses were conducted to identify the factors that were statistically significantly associated with the difference in WASH score. The factors that were found to be significantly associated with the WASH score were entered into a hierarchical stepwise multiple linear regression model to identify the factors associated with an improved WASH score.

### Ethics

The study was approved by the Scientific Advisory Committee and Institutional Ethics Committee of ICMR-NICED and the Institutional Review Board of Christian Medical College, the central coordinating institution.

## RESULTS

Between November 2017 and December 2019, 5991 children were under active surveillance; 258 children were lost to follow-up during the study period. In the first round of the survey, 4104 households were included and 3906 were surveyed in the second round. Because several families contributed multiple eligible children to the study cohort, the number of households under observation was lower than the number of children enrolled in the study.

The sociodemographic characteristics of the study population are presented in Table 1. We stratified the characteristics by the 2 rounds of the survey. In both rounds, a large proportion of the participating families were nuclear in nature, with a median family size of 5 (interquartile range [IQR], 4–6). Most of the families resided in single-room households, as is normal for the area under study. The median monthly family income was slightly higher in round 2 (median Indian rupee [INR] 9000; IQR, INR 8000–12 000) than in round 1 (median INR, 8000; IQR, INR 7000–10 000). A large proportion of the families resided in houses that were mixed kutcha-pucca in construction. Less than half of the households had a separate kitchen and most cooking activities were conducted inside the house. A very small proportion reported to have backyard poultry rearing or cattle of any kind in both the rounds. For classifying houses, we followed the definition laid down in the Statistical Year Book of the Ministry of Statistics and Program Implementation of the Government of India [16]. A pucca house was a permanent one with walls made of burnt bricks, stones packed with lime or cement, cement, concrete, or packed timber, and roof made of tiles, galvanized corrugated iron sheets, asbestos cement sheet, reinforced brick concrete, or reinforced cement concrete. Kutcha houses had roofs and walls made from less-permanent materials such as unburnt bricks, bamboos, mud, grass, reeds, thatch, loosely packed stones, to name a few.

**Table 1.** Characteristics of Families Surveyed in 2 Survey Rounds During 2018–2019

Characteristics	Round 1, 2018	Round 2, 2019
No. of households surveyed	4104	3906
Family size, median (IQR)	5 (4–6)	5 (4–6)
Years of education, median (IQR)	9 (7–10)	9 (7–10)
Rooms in the household excluding kitchen and bathrooms, median (IQR)	1 (1–2)	1 (1–2)
Monthly family income, INR, median (IQR)	8000 (7000–10 000)	9000 (8000–12 000)
Type of family, n (%)		
Nuclear	2563 (62.45)	2494 (63.85)
Extended/3 generational	610 (14.86)	374 (9.58)
Joint	931 (22.69)	1038 (26.57)
Type of house, n (%)		
Pucca	1502 (36.60)	1308 (33.49)
Mixed	2540 (61.89)	2567 (65.72)
Kutcha	62 (1.51)	31 (0.79)
Houses with separate kitchen, n (%)		
Yes	1676 (40.84)	1324 (33.90)
No	2428 (59.16)	2582 (66.10)
Site of cooking, n (%)		
Inside the house	2079 (50.66)	2168 (55.50)
In separate kitchen	1676 (40.84)	1324 (33.90)
Outside the house	308 (7.50)	322 (8.24)
Both inside and outside the house	41 (1.00)	92 (2.36)
Cooking method, n (%)		
Kerosene	1178 (28.70)	978 (25.04)
Liquefied petroleum gas/gas	2496 (60.82)	2609 (66.79)
Electricity	30 (0.73)	21 (0.54)
Firewood/animal waste/crop residue/saw dust	366 (8.92)	279 (7.14)
Coal	34 (0.83)	19 (0.49)
Households with cattle, n (%)		
Yes	88 (2.14)	85 (2.18)
No	4016 (97.86)	3821 (97.82)
Households with chicken or ducks, n (%)		
Yes	155 (3.78)	138 (3.53)
No	3949 (96.22)	3768 (96.47)

Abbreviation: IQR, interquartile range.

In both the rounds, a very high proportion of the respondents reported having access to improved, safe drinking water source, and details are provided in Table 2. Around 1 in 10 households had piped water in the dwelling; the most common source of safe drinking water supplied by the municipal authorities were shared pipes, which ran into the residential yard or public taps used by a group of households. These were the primary source of drinking water for over 80% of the households in both the rounds of the survey. In the second round, an additional question was asked about continuous water access; less than 6% of households indicated that they had continuous access to drinking water. Most families reported having access to intermittent water supply delivered in a time-limited manner, in multiple time intervals each day. Although fetching water was the primary responsibility of the adult women in the households, fewer women reported fetching water in round 2 (45.31%) than in round 1 (61.06%). A median of 20 minutes was spent in fetching water in both rounds. In both the rounds, approximately 1 in 10 families (13.72% in round 1, 9.65% in round 2) reported using any form of home-based water treatment for purification of drinking water. In the second round of the survey, we enquired about the specific strategy being used; use of water filters (6.81%) was the commonest, followed by boiling (1.66%).

**Table 2.** Drinking Water Source and Management in 2 Survey Rounds During 2018–2019

Characteristics	Round 1, 2018 (n = 4104)	Round 2, 2019 (n = 3906)
Main source of drinking water, n (%)		
Piped water into dwellings	473 (11.53)	412 (10.55)
Piped water into yard/plot	1824 (44.44)	1318 (33.74)
Public tap/standpipe	1660 (40.45)	1865 (47.75)
Tube well/borehole	5 (0.12)	7 (0.18)
Bottled water	93 (2.27)	92 (2.36)
Tanker trucks	43 (1.05)	212 (5.43)
Purifiers (electric)	6 (0.15)	0 (0.0)
Drinking water supply availability, n (%)^a^		
Continuous	…	233 (5.97)
Once a day	…	405 (10.37)
More than once a day	…	3223 (82.51)
More than once a week, but not daily	…	45 (1.15)
Time taken to fetch water every day, min		
Mean (SD)	21.52 (13.15)	21.97 (12.46)
Median (IQR)	20 (15–30)	20 (15–30)
Who fetches water most commonly?, n (%)		
Adult women	2506 (61.06)	1770 (45.31)
Adult men	268 (6.53)	307 (7.86)
Female child <15 y	14 (0.34)	19 (0.49)
Male child <15 y	6 (0.15)	5 (0.13)
No response or not applicable	1310 (31.92)	1805 (46.21)
Any water treatment done at the household level, n (%)		
Yes	563 (13.72)	377 (9.65)
No	3538 (86.21)	3527 (90.30)
Do not know	3 (0.07)	2 (0.05)
Specific practices for water treatment, n (%)^a^		
Boil	…	65 (1.66)
Chlorine tablets or bleaching powder	…	6 (0.15)
Strained through a cloth	…	2 (0.05)
Water filter	…	266 (6.81)
Electric purifier	…	36 (0.92)

Abbreviation: IQR, interquartile range.

^a^This question was not included in the first-year survey.

Table 3 summarizes the sanitation facilities that were available to the study population. Over 95% of the households reported having access to some form of improved, flush, or pour-flush latrine. In the 2 rounds, a very small proportion reported using open defecation or hanging toilets. A majority of the sanitation facilities were shared between a median of 6 (IQR, 2–10) households. More households reported accessing sanitation facilities that were open to the general public in round 1 (12.04%) than in round 2 (4.84%). However, despite the almost universal coverage of the study population with improved and safe sanitation facilities, 14.3% of the households reported risky disposal of children’s stools in the community in round 1. In round 2, this proportion had reduced to 8.3%.

**Table 3.** Sanitation Facilities Access, Use, and Practices in 2 Survey Rounds During 2018–2019

Characteristics	Round 1, 2018 (n = 4104)	Round 2, 2019 (n = 3906)
Type of toilet facility used by household members, n (%)		
Flush/pour flush to piped sewer system	1978 (48.20)	1617 (41.40)
Flush/pour flush to septic tank	2051 (49.98)	2168 (55.50)
Flush/pour flush to pit latrine	44 (1.07)	106 (2.71)
Flush/pour flush to an unknown place/do not know	13 (0.32)	4 (0.10)
Pit latrine with slab	1 (0.02)	0 (0.0)
Hanging toilet	2 (0.05)	10 (0.26)
No facilities/open	15 (0.37)	0 (0.0)
Others	0 (0.0)	1 (0.01)
Toilet facilities shared with other households, n (%)		
Yes	3278 (79.87)	3020 (77.32)
No	811 (19.76)	886 (22.68)
No response	15 (0.37)	0 (0.0)
Number of households sharing a toilet facility		
Mean (SD)	8.04 (8.68)	7.13 (7.05)
Median (IQR)	6 (2–10)	6 (2–10)
Use of shared toilet facilities by general public, n (%)		
Yes	494 (12.04)	189 (4.84)
No	2784 (67.84)	2831 (72.48)
Facilities not shared/not accessible by public	826 (20.13)	886 (22.68)
Methods of disposal of children’s stools, n (%)		
Children use toilets	2994 (72.95)	3051 (78.11)
Stool rinsed into toilets	523 (12.74)	530 (13.57)
Stool rinsed into drains/ditches	296 (7.21)	216 (5.53)
Stool disposed with garbage	256 (6.24)	102 (2.61)
Buried	1 (0.02)	0 (0.0)
Stool disposed in the open environment	28 (0.68)	7 (0.18)
Others	6 (0.15)	0 (0.0)

Abbreviation: IQR, interquartile range.

The reported food hygiene practices are summarized in Table 4. A very high proportion of the respondents reported consuming ready-to-eat food from street vendors on a regular basis in both rounds. Over 41% of respondent households in round 1 and 47% in round 2 reported consuming such foods at a frequency greater than or equal to once a week. Similar proportions of the study population reported consuming breakfast or ice creams from such street vendors in both the rounds. However, over half of the respondents in both the rounds reported rarely or never eating uncooked foods, and almost all respondents reported washing such uncooked foods or peeling them before consumption.

**Table 4.** Food Hygiene Practices Surveyed in 2 Survey Rounds During 2018–2019

Characteristics	Round 1, 2018 (n = 4104)	Round 2, 2019 (n = 3906)
Frequency of buying ready to eat food from street vendors, n (%)		
Every day	1029 (25.07)	939 (24.04)
Once a week	770 (18.76)	907 (23.22)
Once a fortnight	147 (3.58)	267 (6.84)
Once a month	202 (4.92)	354 (9.06)
Rarely	1297 (31.60)	1298 (33.23)
Never	659 (16.06)	141 (3.61)
Frequency of eating breakfast from street vendors, n (%)		
Every day	1343 (32.72)	1254 (32.10)
Once a week	635 (15.47)	744 (19.05)
Once a fortnight	77 (1.88)	173 (4.43)
Once a month	65 (1.58)	205 (5.25)
Rarely	1325 (32.29)	1446 (37.02)
Never	659 (16.06)	84 (2.15)
Frequency of eating uncooked food, n (%)		
Every day	821 (20.00)	481 (12.31)
Once a week	668 (16.28)	587 (15.03)
Once a fortnight	79 (1.92)	193 (4.94)
Once a month	118 (2.88)	111 (2.84)
Rarely	1367 (33.31)	1835 (46.98)
Never	1051 (25.61)	699 (17.90)
Washing of uncooked food before eating, n (%)		
Yes	3836 (93.47)	3821 (97.82)
No	268 (6.53)	85 (2.18)
Peeling skin of uncooked food before eating, n (%)		
Yes	3078 (75.00)	3521 (90.14)
No	1026 (25.00)	385 (9.86)
Frequency of consuming ice cream from street vendors, n (%)		
Every day	1238 (30.17)	1337 (34.23)
Once a week	1077 (26.24)	1133 (29.01)
Once a fortnight	178 (4.34)	257 (6.58)
Once a month	282 (6.87)	236 (6.04)
Rarely	1176 (28.65)	908 (23.25)
Never	153 (3.73)	35 (0.90)

**Figure 1.**
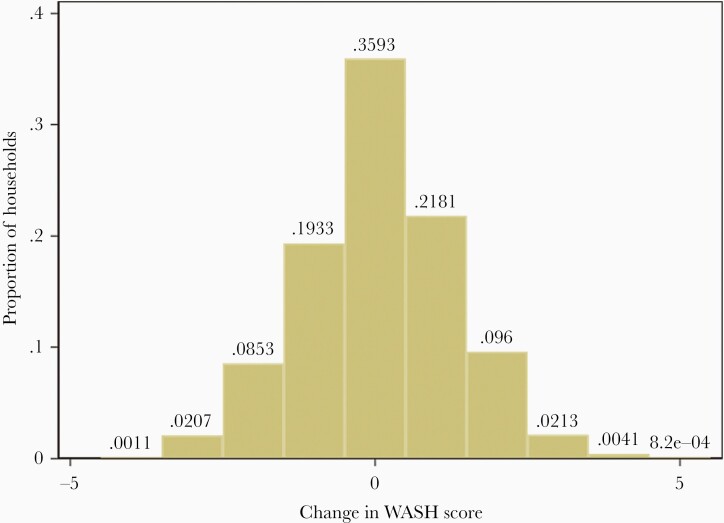
Distribution of water, sanitation, and hygiene (WASH) scores in round 1 (2018) and round 2 (2019) surveys.

A composite WASH score was developed using the reported practices in the 3 domains of water, sanitation, and food hygiene, as outlined in Table 5. Presence of any of the factors mentioned within each of the 3 categories would accrue a point. The differences in the WASH score across the study households in the 2 rounds are summarized in Figure 1 and the distribution of the WASH score in the 2 rounds is provided in Supplementary Table 1. None of the households reported achieving the lowest possible score of 0 in either of the 2 rounds. Only about a third of the households noted an improvement in the WASH score in round 2, with a third noting a reduction in WASH score, and a third showing no change. As noted in Table 5, the maximum possible score on this scale was 10. No households reported receiving the highest WASH score in round 1 and only 1 household reported this score in round 2.

**Table 5.** Indicators of Water, Sanitation and Hygiene Practices Used to Develop Composite WASH Score

Drinking Water Score	Sanitation Score	Food Hygiene Score
Improved source of drinking water	Use of an improved toilet facility	Washing of uncooked food items before consumption
	Toilet is not shared between multiple households	Peeling of skin of uncooked food items before consumption
Safe treatment of drinking water at household level	Toilet is not shared with the general public	Frequent (once a week or more) consumption of street food
	Stool of children is disposed safely by household	Frequent (once a week or more) consumption of ice-cream from street vendors
Possible score: 0 to 2	Possible score: 0 to 4	Possible score: 0 to 4

Unadjusted simple linear regression indicated that the baseline family size, completed years of education, monthly income, housing type, presence of a separate kitchen in the house, location of cooking, cooking methods used, minutes spent fetching water every day, and number of households sharing a toilet were significantly associated with the WASH score (Table 6).

**Table 6.** Results of Univariable Linear Regression Showing Unadjusted Regression Coefficients for Different Factors

Factors	Unadjusted Regression Coefficient (95% CI)	*P* Value
Family size	0.036 (.011 to .061)	.005
Completed years of education	−0.033 (−.046 to −.02)	<.001
Monthly Income, per 10 000	−0.046 (−.121 to .029)	.231
Family type		
Nuclear	Ref	
Extended or 3-generations	−0.068 (−.185 to .048)	.25
Joint	0.153 (.052 to .255)	.003
House type		
Pucca house	Ref	
Mixed Kutcha-Pucca house	0.034 (−.052 to .120)	.435
Kutcha house	−0.283 (−.626 to .059)	.105
Presence of separate kitchen in house	0.105 (.021 to .189)	.015
Location of cooking		
Inside the house	Ref	
Separate kitchen	−0.079 (−.166 to .008)	.074
Outside the kitchen area	0.267 (.108 to .425)	.001
Both inside and outside house	−0.501 (−.908 to −.094)	.016
Cooking methods used		
Kerosene	Ref	
Gas	−0.248 (−.342 to −.155)	<.001
Electricity	−0.063 (−.547 to .420)	.798
Firewood/animal waste/crop residue/saw dust	0.05 (−.107 to .207)	.532
Coal	0.047 (−.405 to .499)	.84
Cattle ownership	−0.012 (−.296 to .271)	.932
Poultry ownership	0.258 (.040 to .477)	.02
Minutes spent in fetching water every day	0.008 (.004 to .013)	<.001
Number of households sharing a toilet	0.022 (.017 to .026)	<.001
Type of toilet facility accessed by household		
Piped sewer system	Ref	
Septic tank	0.401 (.319 to .483)	<.001
Pit latrine	0.00005 (−.388 to .388)	.99
Improved pit latrine	1.646 (.934 to 2.358)	<.001
Hanging toilet	−0.354 (−2.091 to 1.384)	.69
No facilities/field/open defecation	0.289 (−.37 to .948)	.39
Public can access the toilet facilities used by the household	0.017 (.006 to .027)	.001

Abbreviation: CI, confidence interval; Ref, reference.

These variables were then systematically entered into a hierarchical multivariable linear regression model. In the first model, we included the key proximate factors associated with a lower WASH score: years of completed education, family size, number of households sharing a toilet facility, minutes spent in fetching water every day, and public access to toilet used by the household. In the second model, we further added the variables related to housing, including poultry rearing and presence of a separate kitchen. The *f* test for change in *R*^2^ indicated that there was a significant change (*P* = .043) from model 1 and model 2 was a better fit. We then added the variables of location of cooking and family type to construct model 3, but we observed that the change in *R*^2^ was not statistically significant, indicating that model 2 is a better, more parsimonious fit than model 3. Table 7 provides the detailed information on each model. After adjusting for the years of completed education, family size, and public access to family’s toilet facilities, model 2 indicated that there was a significant improvement in WASH score when fewer households shared a toilet and less time was spent in collecting water. However, the model characteristics show a low *R*^2^ value overall, which indicates that other explanatory factors, which were not observed as part of this study, could be associated with improved WASH scores in the households of urban slums in Kolkata.

**Table 7.** Hierarchical Linear Regression Analysis of Factors Associated With WASH Score

Factor	Model 1		Model 2		Model 3	
	β (95% CI)	*P* Value	β (95% CI)	*P* Value	β (95% CI)	*P* Value
Years of completed education	−0.022 (−.042 to −.002)	.033	−0.018 (−.038 to .003)	.086	−0.016 (−.037 to .005)	.129
Family size	0.042 (.004 to .079)	.03	0.043 (.005 to .081)	.026	0.027 (−.018 to .072)	.241
Number of households sharing a toilet	0.021 (.014 to .028)	<.001	0.02 (.013 to .027)	<.001	0.02 (.013 to .026)	<.001
Minutes spent in fetching water every day	0.007 (.003 to .012)	.002	0.006 (.001 to .011)	.01	0.006 (.001 to .01)	.016
Public can access the toilet facilities used by the household	0.006 (−.01 to .022)	.449	0.003 (−.013 to .019)	.733	0.003 (−.014 to .019)	.759
Poultry ownership			0.272 (−.052 to .6)	.1	0.276 (−.047 to .6)	.094
Presence of separate kitchen in house			0.143 (−.001 to .287)	.052	0.204 (.047 to .361)	.011
Location of cooking					0.083 (−.007 to .173)	.071
Family type					0.051 (−.036 to .138)	.252
*R* ^2^	0.05		0.054		0.057	
*R* ^2^ change	…		0.004		0.003	
*P* value for *R*^2^ change	…		0.043		0.088	

Abbreviations: β, adjusted regression coefficient; 95% CI: 95% confidence interval.

## Discussion

Safe WASH practices prevent the transmission of water-borne infections in the community. Our findings mirror those of the National Family Health Survey-4 (NFHS-4), which indicated that 96% of families in the region had access to improved sources of drinking water. Notably, almost all families reported receiving intermittent water supply, and often, during scarcity of water—especially during summer months—local municipal authorities also deploy tanker trucks to meet water needs. A majority of the families believed that the water obtained is safe for drinking, and thus only about one-tenth of the households treated the water in any method before drinking. This is similar to a finding from a study conducted in urban slums of south Delhi [17], where a majority (75%) did not treat the water before drinking, as most of them thought the water was already clean and ready to drink. Almost all the households in the study area had been using a flush toilet, which is much higher than what was reported by NFHS-4. Again, it is worth mentioning that a large proportion had to share their toilet facility with many other local families.

Despite the high degree of coverage with adequate WASH facilities, we observe that most of the households failed to obtain a high WASH score on the composite summary score devised for this study. Although the generalizability of such a score may be in doubt, its internal reliability is assured because all families were measured on the same scale, using uniform methods for data elicitation. This low WASH score is indicative of the vulnerabilities in this area, especially affecting the health of children younger than 5 years. Previous surveys have found that slum-dwelling children younger than 5 years in Kolkata were 3.7 times more likely to suffer from diarrhea than any other age groups [18]. Cross-sectional surveys showed around 8% of children reporting diarrhea in the 2 weeks preceding the survey [19]. Another longitudinal survey showed that children younger than 2 years and between 2 and 5 years reported the highest levels of diarrhea as well as the specific diagnosis of cholera; the incidence of diarrhea in children younger than 2 years was over 270 cases per 1000 person-years, which was almost 5 times higher than the overall incidence rate of 58 cases per 1000 person-years [20]. As a result of these high levels of morbidity, there are long-term implications for children’s health. Surveys have noted high levels of stunting in children from urban slums of Kolkata. One estimate reported as many as 26% of children to be suffering from stunting [21]; another survey reported that 31% were underweight, and amongst them, 29% were stunted and 29% were wasted [22]. In yet another survey, 55% of children younger than 5 years were found to have anthropometric failure, with 48% reporting some form of additional comorbidity, the commonest of which were diarrhea (11%) and acute respiratory illness (9%) [23].

A considerable proportion of this burden of disease and ill health could be averted by improvement in the quality of drinking water along with access to improved sanitation and hygiene facilities in underserved locations. The introduction of clean water, well-designed sewerage systems, and adoption of hygienic practices, along with increased awareness in high-income countries, has led to a dramatic reduction of morbidity and mortality associated with fecal-oral transmission in these countries [5, 24]. Also of interest is the fact that a large proportion of the study population do not undertake any method to purify drinking water. Although drinking water with sufficient residual chlorine is obtained from improved sources by these households, there remains the possibility that the water can be contaminated during storage for drinking, cooking, and other domestic use, as has been previously documented in similar urban slum settings [9].

The lower education level of the head of the household was significantly associated with poor WASH scores, indicating an improvement in WASH practices with increment in education. Similarly, a study conducted among households with children younger than 5 years of the Sugali Tribe of Chittoor, Andhra Pradesh, stated that parents’ higher levels of education were associated with improved WASH practices [25]. Another study conducted among slum-dwellers in Hyderabad, Andhra Pradesh reported that better caregivers’ knowledge was associated with higher odds of improved child hygiene practices [26]. Education level has a practical bearing on all the aspects of living, including hygiene, as this empowers one to accept and practice modern ideas, changing traditional beliefs, attitudes, practices, and augmenting WASH-related knowledge and perspective. However, the gaps between the need for, and access to, health care information and behavior modification still need to be considered in assessing the potential impact of education and awareness-oriented interventions [27].

Almost all of the study population was living in overcrowded conditions in these urban slums (bustees). Most of these families lived in rented structures with extended family members either in single or double rooms, sharing toilets and other necessary infrastructure. Historically, overcrowding has been associated with the spread of infectious diseases. Living in overcrowded households, that is an increase in family size, was significantly associated with unsatisfactory WASH practices in the current study. As reported in a WHO bulletin, a larger family incurs a disproportionately higher cost for practicing safe WASH habits like boiling water or adopting any other purification methods [28]. It is to be noted that overcrowded living situations impose a considerable strain on existing WASH facilities, and with a higher degree of usage and fewer breaks for maintenance, the infrastructure can weaken over time.

It must be noted that as an outcome of the Swachh Bharat Abhiyan, there has been major strides made in coverage of vulnerable areas with appropriate WASH services. Ensured access to a sanitary flush toilet has become a reality, although other essential infrastructure has failed to keep pace over the years, leading to poor drainage in the area, resulting in waterlogging during the monsoon months, along with fecal contamination of the piped water supply. This becomes an especial cause of concern because 1 in 10 families have reported disposing the stool of their children in such open environments, amplifying the threat of diseases that are fecal-oral transmitted. Further, the food safety risks of the area are significant, because a considerable number of children of these families reported frequent consumption of food, including breakfast, from street vendors. It needs to be emphasized that food prepared and sold by the local street vendors, especially in the slum areas, may propagate water-borne infections, owing to the lack of basic hygienic practices of the handlers while preparing and storing food.

There are multiple limitations to our enquiry. There is a chance of recall bias around the yearly data collection timeline. Information on hand hygiene, which is an important part of the WASH practices, was not collected directly in this survey. All data were self-reported, which increases the risk of social desirability bias. Further, there were no observational aspects of the study, which limits the possibility of verifying the reported practices. Thus, we cannot demonstrate the gap between the knowledge and practice in our current effort. In addition, because we depended on self-reported information, there was no scope to evaluate the cleanliness, hygiene, and appropriate use of sanitation facilities. Given the cultural mores around toilet use, qualitative participatory research methods could be employed in future efforts to study these specific attributes. Given the post hoc nature of the analysis and the limitations associated with such approaches, we lay more emphasis on the descriptive findings. Finally, we are conservative in interpreting the identified associations given these limitations, and our inability to ascribe temporality. However, the data for the present analysis were collected as part of a large, community-based surveillance for enteric fever, using well-trained data collectors and field staff, making it highly accurate and internally valid. The data were also scrutinized at multiple time points, ensuring high quality of the data as well as fidelity in the data collection processes. Further, this data will contribute to a deeper understanding of the epidemiology and burden of enteric fever in the community settings, as part of the larger NSSEFI study.

## CONCLUSION

The current analysis finds that despite a high coverage of WASH services in the urban slums of Kolkata, there are gaps in the WASH practices in the population. Although there are limitations in a self-reported, cross-sectional, observational study design, we have noted significant gaps in behavioral patterns, which have remained in spite of the intense focus on improving WASH through the activities of Swachh Bharat Abhiyan in the country. The findings are indicative of the fact that, in addition to providing high-quality WASH services and facilities, there is a need to deploy awareness building and social mobilizing activities, which emphasize building community ownership of these programs. Considering that most of the WASH facilities are currently shared between multiple families, they are unlikely to be used in a safe and sustainable manner unless there is sustainable change in behavioral practices of the community, including contributing to the upkeep, maintenance, and upgrading of such community-owned facilities. More studies are needed to generate evidence useful for planning the most effective intervention to ensure appropriate hygiene practices in the community.

## Supplementary Data

Supplementary materials are available at *The Journal of Infectious Diseases* online. Consisting of data provided by the authors to benefit the reader, the posted materials are not copyedited and are the sole responsibility of the authors, so questions or comments should be addressed to the corresponding author.

jiab354_suppl_Supplementary_Table_1

## Notes

***Acknowledgments*.** We are thankful to all the participants, project staff, and the community involved in the study. We are also thankful to the Central Coordinating team from Christian Medical College, Vellore for structuring and monitoring the data collection process.

***Author contributions.*** S. K., P. C., J. S., and N. C. collected the demographic and clinical data. S. K. processed the data and conducted the statistical analysis. S. K., P. C., and T. P. drafted the manuscript. S. D. revised the final manuscript. All the authors have read and approved the final version. The corresponding author had full access to all the data in the study and had final responsibility for the decision to submit for publication.

***Disclaimer***. The funder of the study had no role in study design, data collection, data analysis, data interpretation, or writing of the report.

***Financial support*.** The work was supported by the Bill and Melinda Gates Foundation (grant number INV-009497-OPP1159351) through the Christine Medical College, Vellore.

***Supplement sponsorship.*** This supplement is sponsored by the Christian Medical College Vellore Association.

***Potential conflicts of interest*.** All authors: No reported conflicts of interest. All authors have submitted the ICMJE Form for Disclosure of Potential Conflicts of Interest. Conflicts that the editors consider relevant to the content of the manuscript have been disclosed.

Presented in part: 11th International Conference on Typhoid and Other Invasive Salmonelloses, Hanoi, Vietnam, 26–28 March 2019.
