# Supplementary material for: Water, Sanitation, and Hygiene Practices in Urban Slums of Eastern India
Source: J Infect Dis. 2021 Nov 23;224(Suppl 5):S573–83. doi: 10.1093/infdis/jiab354 (PMC8892530; doi:10.1093/infdis/jiab354)
Supplement: jiab354_suppl_Supplementary_Table_1 [file jiab354_suppl_supplementary_table_1.docx]

**Supplementary Table 1.** Distribution of WASH Scores across households by the rounds of survey

| **WASH Score** | **Round 1 (n, %)** | **Round 2 (n, %)** |
| --- | --- | --- |
| **3** | 13 (0.32) | 1 (0.03) |
| **4** | 118 (2.88) | 26 (0.67) |
| **5** | 494 (12.04) | 331 (8.47) |
| **6** | 1,256 (30.6) | 1,473 (37.71) |
| **7** | 1,201 (29.26) | 1,106 (28.32) |
| **8** | 876 (21.35) | 865 (22.15) |
| **9** | 146 (3.56) | 103 (2.64) |
| **10** | 0 (0.0) | 1 (0.03) |
